# Ultratrace eNose Sensing of VOCs toward Breath Analysis Applications Utilizing an eNose-Based Analyzer

**DOI:** 10.1021/acsmeasuresciau.3c00053

**Published:** 2024-02-05

**Authors:** Johannes Glöckler, Carsten Jaeschke, Marta Padilla, Jan Mitrovics, Boris Mizaikoff

**Affiliations:** † Institute of Analytical and Bioanalytical Chemistry, Ulm University, Albert-Einstein-Allee 11, 89081 Ulm, Germany; ‡ JLM Innovation GmbH, Vor dem Kreuzberg 17, 72070 Tübingen, Germany; § Hahn-Schickard, Sedanstrasse 14, 89077 Ulm, Germany

**Keywords:** eNose, metal oxide sensors, MOX, sensor array, VOCs, principal component analysis, breath analysis

## Abstract

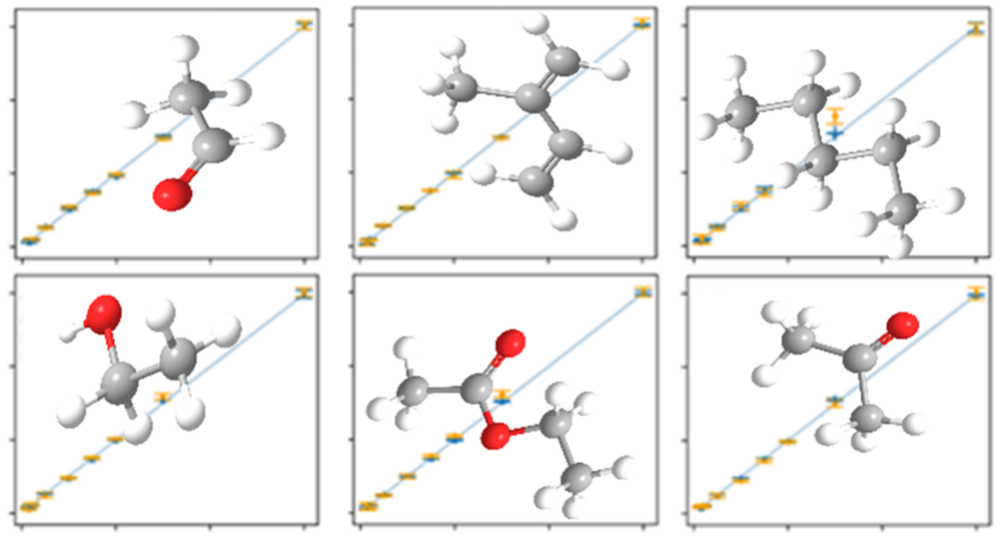

This proof-of-principle study presents the ability of the recently developed iLovEnose to measure ultratrace levels of volatile organic compounds (VOCs) in simulated human breath based on the combination of multiple gas sensors. The iLovEnose was developed by our research team as a test bed for gas sensors that can be hosted in three serially connected compact low-volume and temperature-controlled compartments. Herein, the eNose system was equipped with conventional semiconducting metal oxide (MOX) gas sensors using a variety of base technologies providing 11 different sensor signals that were evaluated to determine six VOCs of interest at eight low to ultralow concentration levels (i.e., ranging from 3 to 0.075 ppm) at humid conditions (90% rh at 22 °C). The measurements were randomized and performed four times over a period of 2 weeks. Partial least-squares regression analysis was applied to estimate the concentration of these six analytes. It was shown that the iLovEnose system is able to discriminate between these VOCs and provide reliable quantitative information relevant for future applications in exhaled breath analysis as a diagnostic disease detection or monitoring device.

With the increasing worldwide incidence of chronic diseases and the occurrence of emerging diseases (e.g., COVID-19, long COVID, etc.), the development of POC devices that are able to detect molecular biomarkers in a sensitive, noninvasive, and low-cost manner for screening and monitoring of diseases has gained relevance. In this regard, more than 1000 compounds have been identified to be present in exhaled human breath at concentrations ranging from parts per billion to parts per trillion levels. Approximately 35 of the identified compounds in exhaled breath have been established as biomarkers for particular diseases and metabolic disorders. In the present study, six of the most common biomarkers reported in exhaled breath of patients with diseases such as lung cancer, breast cancer, and COPD—among others—were considered.^1−13^

The ultratrace sensing was performed using the recently presented eNose system,^14^ the so-called “modular integrated-low-volume-eNose (iLovEnose)”^15^ (Figure S-1), an eNose designed for online measuring purposes that can be used as add-on to routine testing. The iLovEnose system consists of three exchangeable individual compartments (or modules) that can host different sensors and different sensor types (analogue, digital, and experimental sensors, etc.). These three serially connected compartments share a compact, low-volume (less than 3 mL), and temperature-controlled (set to 45 °C) sensing chamber.^14,16^

Here, the utilized iLovEnose setup of the three-slot sensing chamber contains one compartment with eight analog and two compartments with 10 digital MOX sensors, suitable for different target gases (Table S-1).^14,15^

The measurements of different VOCs of interest in the field of breath gas analysis (acetaldehyde, acetone, ethanol, ethyl acetate, isoprene, and *n*-pentane) were performed at eight low to ultralow concentration levels (3, 1.5, 1, 0.75, 0.5, 0.25, 0.1, and 0.075 ppm) under humid conditions (90% rh @ 22 °C) to demonstrate the possible use case of VOC detection in human breath. The objective of the experiment is to discriminate between the different VOCs and derive an estimation of their ultralow concentrations.

The experimental setup for humidified measurements is schematically shown in Figure S-2. For humidified measurements, the gas mixing system (GMS) utilizes a set of mass flow controllers (MFCs) (manufacturer: Bronkhorst High-Tech B.V., AK Ruurlo, The Netherlands) and a humidity generator (manufacturer: Owlstone, Cambridge, UK). The resulting gas flows first through a grid (mesh size 15 μm; manufacturer: Swagelok, Solon, Ohio, USA) to create a homogeneous VOC mixture and then through a dew point meter (manufacturer: Michell Instruments, Cambridge, UK) to measure the humidity content of the generated gas mixture. The certified VOC gas standards used (nominal concentration: 200 ppm in synthetic air; vendor: Westfalen AG, Weißenhorn, Germany) were diluted with hydrocarbon-free synthetic air (nominal concentration: 20.5% O_2_ in N_2_; vendor: MTI IndustrieGase AG, Neu-Ulm, Germany) to the concentration range of 75 ppb to 3 ppm (in eight concentration steps). A custom-made mass-flow-controlled GMS with LabVIEW-based software was used to run the automated flow schedule. The diluted gases were then fed into our iLovEnose system with a total flow rate of 800 mL/min.

The flow of the humidity generator was permanently set to 700 mL/min to achieve a constant humidity level. The water content was measured additionally with BME680 sensors inside the sensing chamber and was 22.02 ± 1% rh @ 45 ± 1 °C, resulting in a water content of air approximately 16.44 g m^–3^.

All eight concentration steps from 0.075 to 3 ppm (0.075, 0.1, 0.25, 0.5, 0.75, 1, 1.5, and 3 ppm) were randomized and applied with the six selected VOCs of interest. The measurements were performed four times over a period of 2 weeks. The general flow schedule is shown in Figure S-3.

For each measurement, after the 10 min of exposure to a target VOC, clean air flows through the device for 10 min to clean the system and recover the sensor.

The objective of the experiment is to estimate the concentration of the six analytes using partial least-squares (PLS).^17^ Analysis was performed using Python3^18^ and the packages scikit-learn^19^ and plotnine^20^ for plotting. The used data set contains a total of 354 measurements of six analytes at eight concentration levels in synthetic air under humid conditions (90% rh @ 22 °C).

Although the eNose is designed to host up to 36 sensors distributed in the three modules, in this work only 16 sensor signals originating from nine sensor modules were used, which are summarized in Table S-2. The signals of the redundant sensors in each compartment were averaged so that one signal per sensor type is considered in the data. That gives 11 different sensor signals for the analysis. Additionally, a few samples corresponding to first measurements of the day were removed from the data set. These outliers were present because the sensors were not hot enough at the beginning of the measurement set. Thus, the complete data set composition is shown in Table S-3.

All of the sensor signals were recorded during the whole measurement at a 1 Hz sampling rate. However, in this analysis, only one point was considered from each sensor per measurement. Such a point corresponds to an average of 5 s before the cleaning step, i.e., the sensor’s steady state. Similarly, for each sensor an average of 5 s before the analyte exposure was taken as baseline. Then a preprocessing step computed *x*_*i*_ = *r*_o*i*_/*r*, where *r*_o*i*_ denotes the baseline resistance and *r* the sensor resistance response to the analyte at its steady state. Later, matrix **X** containing the sensor data was autoscaled, and then a dimensionality reduction step using principal component analysis (PCA) was performed. Finally, PLS^17^ was the regression method applied to estimate the concentration levels of the different analytes.

The chosen validation strategy was three-way-split cross-validation (CV). The data set was split into three subsets: training, validation, and test sets. CV with different training and validation sets allows the best model to be found among different parameter values, which consist of combinations of the dimensionality reduction parameter, given by PCA’s number of principal components (PCs) or PCA-explained variance, and the number of latent variables (LVs) for PLS regression. Specifically, the tested parameters are the PCA percentage of explained variance {90, 95, 98, 99, 99.9} and the PLS number of LVs {2 to 11}. The final evaluation was made over the test set, whose samples had never been used for training or validation.

To evaluate the final predictions, three scores were computed: mean absolute error (MAE), root-mean-square error (RMSE), and the coefficient of determination (*R*^2^):
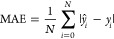

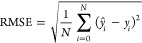

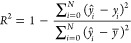
where *N* is the number of samples, *ŷ*_*i*_ is the estimated concentration of sample *i*, *y*_*i*_ is the known concentration level of sample *i*, and *y̅* is the mean of the data concentration levels *y*.

Table 1 shows the results of the applied methodology to estimate the concentration levels of the six analytes. The three score functions are shown per data subset (training, validation, and test) along with the best set of parameter values found using the validation subset (number of PCs or explained variance using PCA and number of LVs for regression using PLS). A graphical comparison of the predicted and real concentration values obtained for one data partition into training and test subsets is shown in Figure 1 for each of the six analytes. Numerical errors for this particular run are shown in each of the plots.

**Figure 1:**
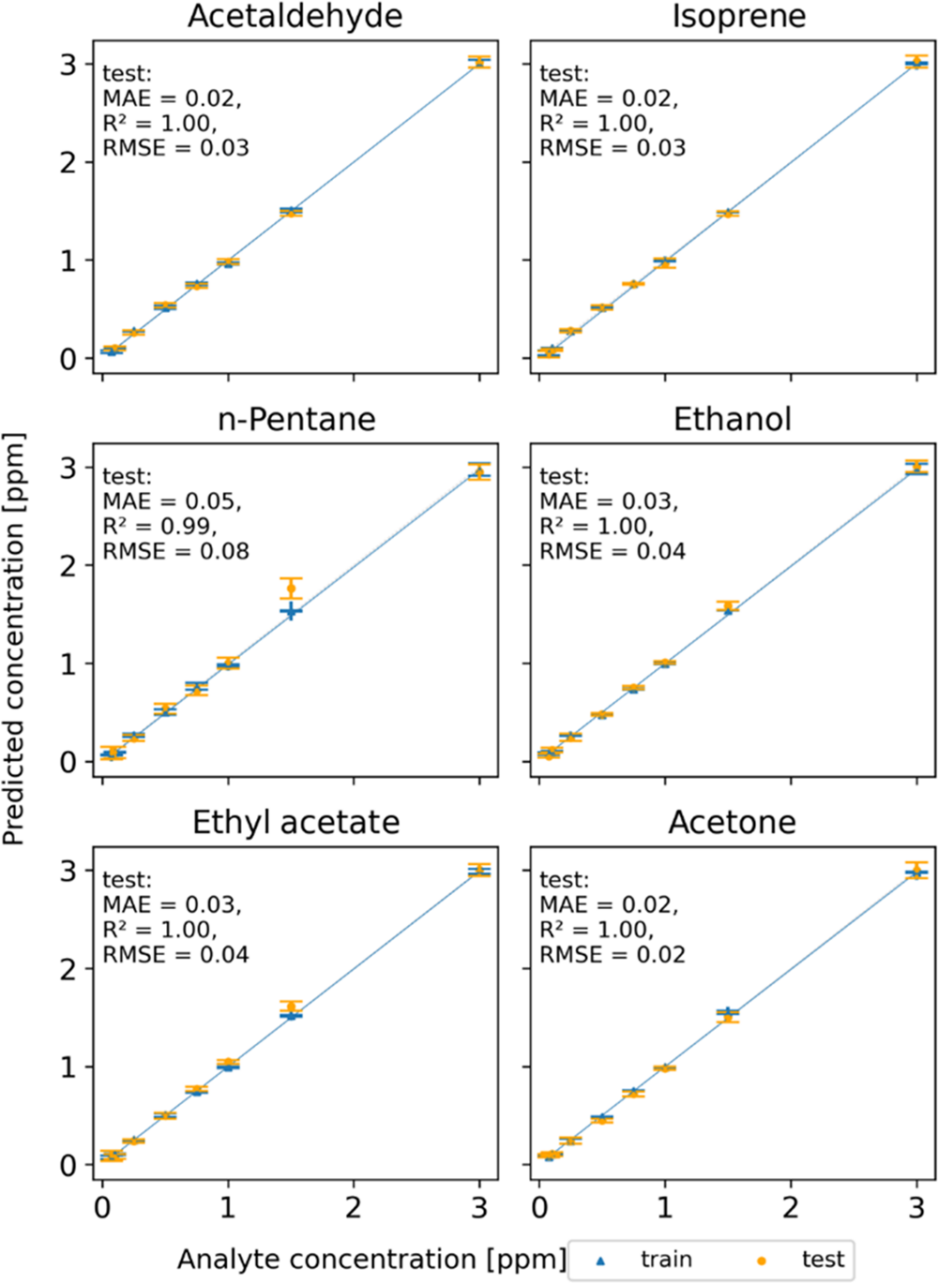
Results of one particular CV data partition into training and test sets for each analyte using the best PCA+PLS estimator found, for which the numbers of PCs and LVs are specified in Table 1a. The numbers of pairs in the training and test sets are specified in Table S-4.

**Table 1:** PCA+PLS Results for Experiments under Humidity Conditions

(a) Training Set						
	PCA		PLS		training set mean ± σ	
analyte	var [%]	no. of PCs	no. of LVs	MAE [ppm]	RMSE [ppm]	*R*^2^
acetaldehyde	99.9	6	6	0.014 ± 0.001	0.019 ± 0.001	1.0 ± 0.0
acetone	99.9	5	5	0.011 ± 0.001	0.016 ± 0.001	1.0 ± 0.0
ethanol	99.9	5	5	0.02 ± 0.002	0.026 ± 0.002	0.999 ± 0.0
ethyl acetate	99.9	6	6	0.012 ± 0.003	0.016 ± 0.003	1.0 ± 0.0
isoprene	99.9	5	4	0.021 ± 0.002	0.031 ± 0.003	0.999 ± 0.0
*n*-pentane	99.9	6	6	0.012 ± 0.001	0.015 ± 0.001	1.0 ± 0.0

(b) Validation and Test Sets						
	validation set mean ± σ			test set		
analyte	MAE [ppm]	RMSE [ppm]	*R*^2^	MAE [ppm]	RMSE [ppm][ppm]	*R*^2^
acetaldehyde	0.018 ± 0.008	0.025 ± 0.011	0.999 ± 0.002	0.018	0.025	0.998
acetone	0.014 ± 0.006	0.018 ± 0.008	0.999 ± 0.001	0.013	0.017	0.999
ethanol	0.026 ± 0.01	0.034 ± 0.016	0.997 ± 0.003	0.026	0.035	0.996
ethyl acetate	0.017 ± 0.007	0.021 ± 0.009	0.999 ± 0.002	0.014	0.018	0.999
isoprene	0.026 ± 0.011	0.035 ± 0.017	0.997 ± 0.004	0.025	0.035	0.996
*n*-pentane	0.016 ± 0.005	0.02 ± 0.006	0.999 ± 0.001	0.015	0.019	0.999

The data analysis of the experiment for the six analytes under humid conditions (Table 1) shows errors (MAE and RMSE) which are much lower than the smallest concentration level and *R*^2^ values which are high, lying around 0.99. Errors below 0.0375 ppm (0.075/2 ppm as half of the lowest concentration) are colored red in the result tables for the test set. The obvious decrease in performance between the training and test sets can also be observed, but the errors are only slightly worse for the test set with respect to the training set.

A significant correlation among the sensors is also shown in Table 1, since 99.9% of the explained variance can be reached with five or six of the 11 sensor signals used. In addition, we can see that PLS needs four to six LVs. The number of LVs needed is limited by the reduced number of components: it is high whenever the number of PCs is high. Since PLS is a linear method, the high number of LVs for a single analyte indicates that nonlinearities are present in the sensor data with relation to the concentration values. These nonlinearities are similar for all six analytes, as the LVs (and also PCs) do not have a large variation among the analytes.

Errors for ethanol and isoprene are, in general, slightly higher than for the other analytes, although according to Figure 1 the worst-performing case is *n*-pentane. This is because it corresponds to a particular selection of one training set and one test set.

The results for this experiment are promising for applications in the field of breath analysis, although the analytes were individually presented to the eNose. However, low errors were obtained for the tested very low concentration levels with a relatively small training set and using a simple linear PLS regression method.

We are aware of the complexity of real breath analysis applications, such as mixtures of hundreds of compounds, influence of other variables, etc., and the gas sensors themselves, such as drift, sensor-to-sensor variability, etc. However, here we have shown that very low concentrations of several analytes typically present in human breath can be detected with very small error rates by our eNose. Future experiments will be focused on demonstrating the ability of the system to investigate real-world exhaled breath samples in a variety of screening applications.

## Supporting Information Available

List of used sensors with the number of total signals; number of measurements for each analyte and concentration used for the analyses; numbers of samples in the training and test sets; schematic drawings of the used eNose and the experiental setup; general flow schedule of a measurement cycle (PDF)
