# Supplementary material for: Ultratrace eNose Sensing of VOCs toward Breath Analysis Applications Utilizing an eNose-Based Analyzer
Source: ACS Meas Sci Au. 2024 Feb 5;4(2):184–7. doi: 10.1021/acsmeasuresciau.3c00053 (PMC11027196; doi:10.1021/acsmeasuresciau.3c00053)
Supplement: Supplementary file 1 — tg3c00053_si_001.pdf [file tg3c00053_si_001.pdf]

Supporting Information

Ultratrace eNose Sensing of VOCs toward Breath Analysis Applications Utilizing an eNose-Based Analyzer

Johannes Glöckler [1‡], Carsten Jaeschke [1‡], Marta Padilla [2‡], Jan Mitrovics [2] and Boris Mizaikoff [1,3*]

[1] Ulm University, Institute of Analytical and Bioanalytical Chemistry, Albert-Einstein-Allee 11, 89081 Ulm, Germany.

[2] JLM Innovation GmbH, Vor dem Kreuzberg 17, 72070 Tuebingen, Germany.

[3] Hahn-Schickard, Sedanstrasse 14, 89077 Ulm, Germany.

[‡] These authors contributed equally.

* Corresponding author: boris.mizaikoff@uni-ulm.de

Table of Contents

Table S-1. Target gases and stated optimal detection concentration by manufacturer of incorporated sensors.

Table S-2. Sensors and output signals used for data analysis.

Table S-3. Number of measurements giving the dataset content after outlier removal.

Table S-4. Number of samples in corresponding subsets for k-fold CV.

Table S-5. Accuracy of used mass flow controllers.

Figure S-1. Novel modular, integrated-low-volume-eNose (iLovEnose) system based on commercial MOX sensors: (a) fully assembled device; (b) right side and front opened. Reproduced from Jaeschke et al.1 with permission from the Royal Society of Chemistry.

Figure S-2. Schematic drawing of the experimental setup for humid measurements. Reproduced from Jaes-chke et al.1 with permission from the Royal Society of Chemistry

Figure S-3. General flow schedule cycles with two times eight randomized concentration steps.

Figure S-4. Exemplary representation of the signals of the analog sensors during a measurement cycle.

Table S-1. Target gases and stated optimal detection concentration by manufacturer of incorporated sensors.

Type	Sensor Name	Manufacturer	Target Gases	Optimal Detection Concentration [ppm]
Analog	MICS-6814	SGX SENSORTECH Ltd.	Carbon monoxide	1 – 1000
			Nitrogen dioxide	0.05 – 10
			Ethanol	10 – 500
			Hydrogen	1 – 1000
			Ammonia	1 – 500
			Methane	>1000
			Propane	>1000
			Iso-butane	>1000
	MICS-4514	SGX SENSORTECH Ltd.	Carbon monoxide	1 – 1000
			Nitrogen dioxide	0.05 – 10
			Ethanol	10 – 500
			Hydrogen	1 – 1000
			Ammonia	1 – 500
			Methane	>1000
	CCS801	ams Sensor Solutions Germany GmbH	Air contaminants	
			Hydrogen	1 – 30
	TGS8100	FIGARO Engineering, Inc.	Air quality	-
Digital	BME680	Bosch Sensortec GmbH	Air quality	-
	CCS811	ams Sensor Solutions Germany GmbH	Ethanol	0 – 1000
			Hydrogen	0 - 1000
	SGP30	Sensirion AG	Air quality	
			Ethanol	0 – 1000
	ZMOD4410	Integrated Device Technology, Inc.	Air quality	-

Table S-2. Sensors and output signals used for data analysis.

Module	Sensor type	Number of incorporated sensors	Sensor output signals
1	MiCS 6814	1	MICS_6814_NH3<br>MICS_6814_red.<br>MICS_6814_ox.
	TGS 8100	1	TGS_8100
	CCS 801	2	CCS_801_A<br>CCS_801_B
	MiCS 4514	1	MICS_4514_red.<br>MICS_4514_ox.
2	SGP 30	2	SGP_H2_A<br>SGP_H2_B<br>SGP_EtH_A<br>SGP_EtH_B
3	SGP 30	2	SGP_H2_A<br>SGP_H2_B<br>SGP_EtH_A<br>SGP_EtH_B
Total	5	9	16

Table S-3. Number of measurements giving the dataset content after outlier removal.

Analyte	Concentration levels in ppm								Total
	0.075	0.1	0.25	0.5	0.75	1.0	1.5	3.0	
Acetaldehyde	7	7	7	8	8	7	8	7	59
Acetone	7	8	7	7	7	6	7	8	57
Ethanol	8	7	7	8	8	7	8	7	60
Ethyl acetate	8	7	7	8	8	7	8	7	60
Isoprene	8	7	7	8	8	7	8	7	60
n-Pentane	7	8	7	7	7	7	7	8	58

Table S-4. Number of samples in corresponding subsets for k-fold CV.

CV		Dataset	Average range of # samples		
# splits	# repetitions	# samples range	training	validation	test
8	5	56-60	44-47	6-7	6-7

Table S-5. Accuracy of used mass flow controllers.

Product description	Accuracy	Distributor
EL-FLOW Prestige FG-201CV-RBD-11-K-DA-000 30 mL min^{-1}	± 0.5% reading plus ± 0.1% fullscale	Bronkhorst High-Tech B.V. (AK Ruurlo, Netherlands)
EL-FLOW Prestige FG-201CV-RBD-11-K-DA-000 50 mL min^{-1}	± 0.5% reading plus ± 0.1% fullscale	Bronkhorst High-Tech B.V. (AK Ruurlo, Netherlands)v
EL-FLOW Prestige FG-201CV-RBD-11-K-DA-000 1000 mL min^{-1}	± 0.5% reading plus ± 0.1% fullscale	Bronkhorst High-Tech B.V. (AK Ruurlo, Netherlands)
OHG-4 3000 mL min^{-1}	± 0.5% reading plus ± 0.3% fullscale	Owlstone Inc. (Cambridge, United Kingdom)

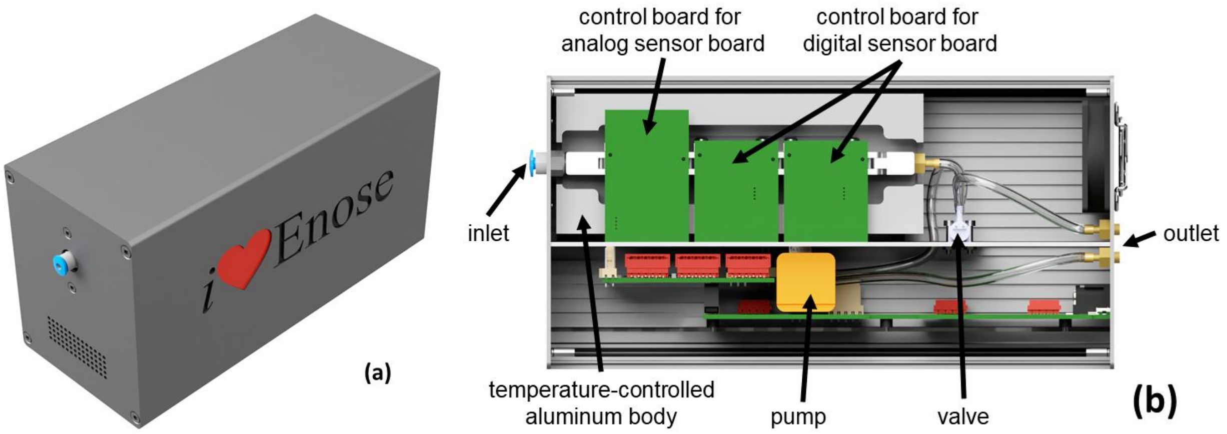

Figure S-1. Novel modular, integrated-low-volume-eNose (iLovEnose) system based on commercial MOX sensors: (a) fully assembled device; (b) right side and front opened. Reproduced from Jaeschke et al.[1] with permission from the Royal Society of Chemistry.

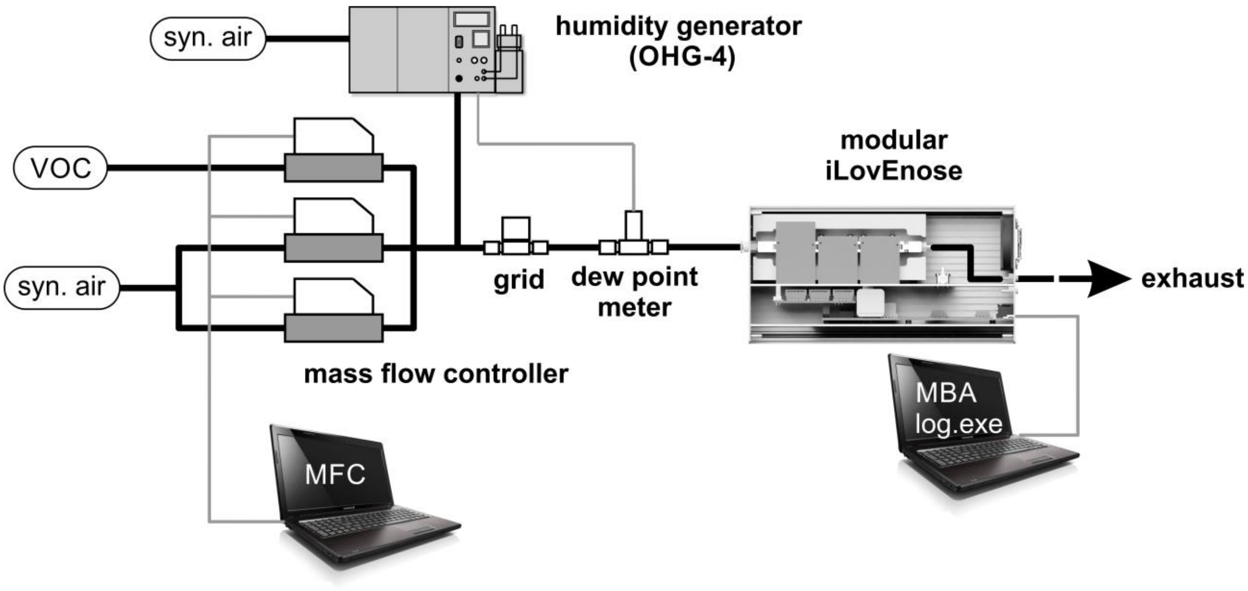

Figure S-2. Schematic drawing of the experimental setup for humid measurements. Reproduced from Jaeschke et al.[1] with permission from the Royal Society of Chemistry.

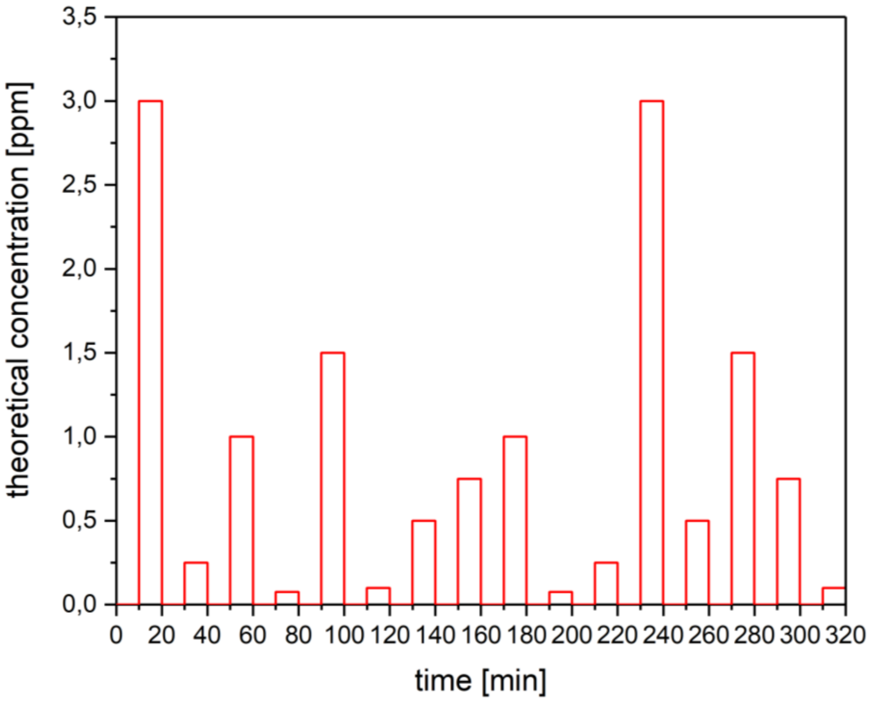

Figure S-3. General flow schedule cycles with two times eight randomized concentration steps.

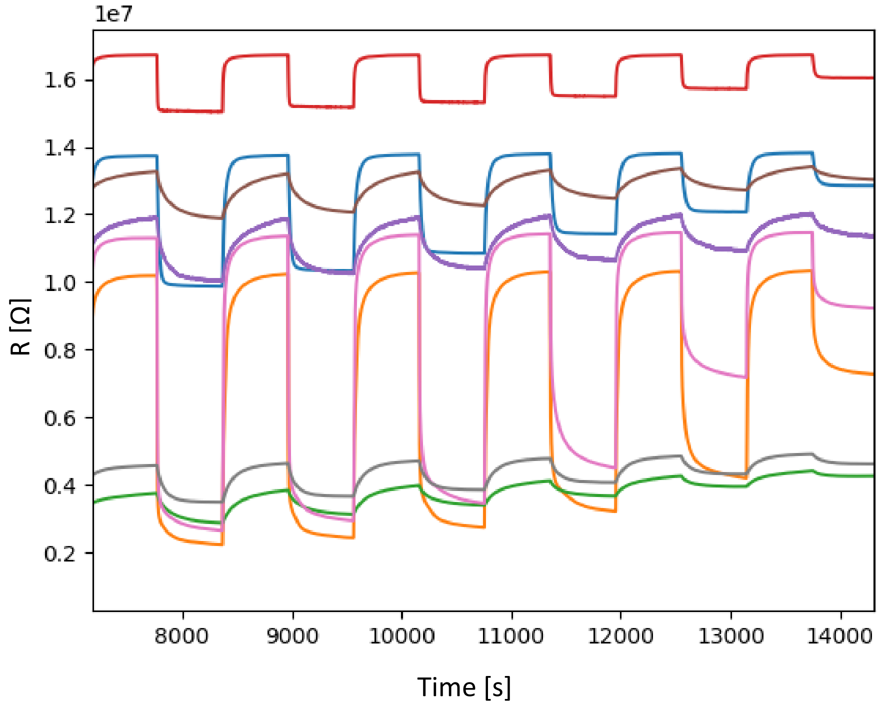

Figure S-4. Exemplary representation of the signals of the analog sensors during a measurement cycle.

References

(1) Jaeschke, C.; Glöckler, J.; Padilla, M.; Mitrovics, J.; Mizaikoff, B. An ENose-Based Method Performing Drift Correction for Online VOC Detection under Dry and Humid Conditions. *Anal. Methods* **2020**, *12* (39), 4724–4733. https://doi.org/10.1039/D0AY01172J.
